# CD97 promotion of gastric carcinoma lymphatic metastasis is exosome dependent

**DOI:** 10.1007/s10120-015-0523-y

**Published:** 2015-08-02

**Authors:** Daren Liu, Chao Li, Bogusz Trojanowicz, Xiaowen Li, Dike Shi, Chenni Zhan, Zhefang Wang, Li Chen

**Affiliations:** Department of General Surgery, Second Affiliated Hospital, College of Medicine, Zhejiang University, 88 Jiefang Street, Hangzhou, 310009 People’s Republic of China; Research Lab, Department of Internal Medicine II, Halle (Saale) University Hospital, Halle (Saale), Germany

**Keywords:** CD97, Gastric cancer, Exosomes, Lymphatic metastasis

## Abstract

**Background:**

CD97 knockdown impairs the metastatic capacity of SGC-7901 gastric cancer cells. However, the role of CD97 in the distant lymphatic premetastatic niche formation of gastric cancer remains unknown.

**Methods:**

Exosomes and the soluble fraction were isolated from SGC-L (an SGC-7901-cell-derived highly lymphatic metastatic cell line) and CD97-knockdown (SGC-L/CD97-kd) cells, and were co-cultured with gastric cancer cells. The metastatic capacity of the two cell lines was evaluated in vitro and in a footpad lymph node metastasis mouse model. Premetastatic-niche-formation-related proteins were examined immunohistochemically.

**Results:**

CD97 expression was ninefold higher in SGC-L cells than in SGC-7901 cells. In vitro, exosomes or conditioned medium from the SGC-L cells enhanced cell proliferation (20 % increase) and invasion (30 % increase) as compared with that from SGC-L/CD97-kd cells (*p* < 0.01). Intrafootpad injections of SGC-L, but not SGC-L/CD97-kd exosomes or conditioned medium, strongly promoted SGC-L and SGC-L/CD97-kd cell accumulation in the draining lymph nodes (*p* < 0.01) and increased CD55, CD44v6, α_5_β_1_, CD31, epithelial cell adhesion molecule, and CD151 expression. Although the SGC-L/CD97-kd exosomes alone were insufficient for promotion of metastasis, they were partly aided by the SGC-L-cell-derived soluble fraction.

**Conclusions:**

The CD97 small isoform promotes SGC-L cell lymphatic metastasis exosome dependently, and aided by the soluble fraction, the exosome-dependent CD97 plays a pivotal role in premetastatic niche formation.

**Electronic supplementary material:**

The online version of this article (doi:10.1007/s10120-015-0523-y) contains supplementary material, which is available to authorized users.

## Introduction

Lymphatic metastasis is the commonest means of gastric carcinoma spread [1]. In metastasis formation, cancer-initiating cells detach from the local tumor and travel to the premetastatic niche in the target organ, organized by long-distance communication [2]. CD97, a member of the epidermal growth factor (EGF)–seven transmembrane subfamily, is overexpressed in most gastric carcinomas, where enhanced CD97 expression in gastric carcinoma is associated with tumor cell dedifferentiation and aggressiveness and directly correlates with clinical pathological parameters such as the tumor–node–metastasis (TNM) classification [3–5]. In most cases, CD97 is only expressed in the primary tumor mass of gastric carcinoma; but in a few cases, especially those with poor differentiation, CD97 expression is enhanced in the regional draining lymph nodes [6]. Elevated CD97 expression and CD44 and CD31 expression were found in the early metastatic regional lymph nodes of an orthotopically implanted gastric cancer mouse model, but their expression was strongly downregulated in a CD97/EGF1,2,5 knockdown group [7], which demonstrated that CD97 not only supports local tumor growth, but also promotes lymph node metastasis.

Exosomes are small membrane vesicles secreted by various cell types, including lymphocytes, epithelial cells, stem cells, and tumor cells [8]. Exosomes not only have membrane components but also contain microRNA (miRNA), messenger RNA (mRNA), and other noncoding RNAs, which can be delivered to recipient cells and internalized, where they can be translated and cause host cell silencing [9]. It was reported that tumor-derived exosomes contribute to metastatic niche formation and promote tumor growth in breast cancer [10]. Capitalizing on their long-distance gene-delivering characteristic, exosomes are considered mediators of the intercellular communication between the local tumor and the premetastatic niche in the host organs, which facilitates gastric carcinoma metastasis by promoting premetastatic niche formation.

We used highly lymphatic metastatic (SGC-L) and poorly lymphatic metastatic (SGC-L/CD97-kd, where “kd” represents “knowdown“) gastric cancer cell lines to prove our hypothesis that CD97 promotes gastric carcinoma lymphatic metastasis through exosome-dependent long-distance cross talk. Additionally, to determine whether other tumor-derived components are involved in premetastatic niche formation, we investigated the impact of the soluble fraction on the metastatic potential of the cells.

## Materials and methods

### Cell lines and animals

The gastric adenocarcinoma cell line SGC-7901 was purchased from ATCC (http://www.atcc.org). The cell line was originally derived from the metastatic lymph node of a patient who had received no prior therapy, and was characterized by pathology and chromosome examination. The cells were propagated in RPMI 1640 medium (Genom Biologic, Hangzhou, China) and 10 % fetal bovine serum (FBS), as previously described [7].

The 4–6-week-old female BALB/c nu/nu mice (18–22 g) used in this study were housed in a sterile environment and maintained under a daily 12-h light/dark cycle, which was controlled by qualified staff at the Zhejiang University Laboratory Animal Center, Hangzhou, China. All animal studies were performed in strict accordance with the guidelines for the welfare and use of animals in cancer research of the Committee of the National Cancer Research Institute. The Zhejiang University Animal Research Committee approved the protocol. All surgical procedures were performed with the mice under sodium pentobarbital anesthesia, and all efforts were made to minimize suffering [11].

### Establishment of footpad lymph node metastasis mouse model and SGC-L cell generation

Twenty BALB/c nu/nu mice were used for tumor implantation. Subconfluent SGC-7901 cells (1.0 × 10^8^/mL) were injected into the footpad of each mouse. After 6–8 weeks, the mice were killed according to institutional guidelines before they developed signs of distress. The draining popliteal and inguinal lymph nodes were harvested for histological examination and primary cell culture [12]. The lymph nodes were minced into 1-mm^3^ pieces and digested with collagenase type II in RPMI 1640 medium. The primary culture metastatic tumor cells were propagated and reinjected into the footpads of the mice. After this process had been repeated four times, when the lymph node metastasis rate was more than 70 %, the subpopulation offspring of the SGC-7901 cells within the lymph nodes, which had higher lymphatic metastatic ability, was preserved and designated SGC-L.

### Cell proliferation assay

Cells (1 × 10^3^ per well) were cultured in the presence of conditioned medium or exosomes. Proliferation was determined at 24, 48, and 72 h by 3-(4,5-dimethylthiazol-2-yl)-5-(3-carboxymethoxyphenyl)-2-(4-sulfophenyl)-2*H*-tetrazolium (MTS) staining [13]. Cells (1 × 10^3^ per well) were plated in 200 µL RPMI 1640 medium with tumor-cell-derived conditioned medium or fractions (exosomes, soluble fraction) in 96-well plates. At 24, 48, and 72 h, the growth medium was replaced with 100 µL serum-free medium, and 20 µL MTS solution (Promega, Madison, WI, USA) was added. The absorbance was measured at 490 nm with a microplate reader (Bio-Rad, Hercules, CA, USA).

### Invasion and scratch wound assays

Invasion assays were performed with 24-well Transwell™ chambers (Costar, MA, USA) with Matrigel-coated 8-μm filters (BD Biosciences, San Jose, CA, USA). Cells (1.0 × 10^5^) in medium containing 1 % FBS were added to the upper chamber; the lower chamber was filled with medium containing 10 % FBS as a chemoattractant. After incubation for 24 h, the transmigrated cells were fixed, stained with 0.2 % crystal violet (Sigma, Munich, Germany), and counted under a light microscope (Leica, Wetzlar, Germany). The scratch wound assay was done with a standard 200-μL pipette tip in 80 % confluent cells. Wound healing was evaluated after 24 h with a light microscope (Leica, Wetzlar, Germany).

### CD97 knockdown by miRNA

Four candidate miRNA sequences targeting human CD97 (Table S1) were designed and cloned into pcDNA6.2-GW/EmGFP-miR vector (Invitrogen, Grand Island, NY, USA). We transfected SGC-L cells with the vectors using Lipofectamine 2000 according to the manufacturer’s protocol (Life Technologies, Grand Island, NY, USA). CD97 silencing was verified by reverse transcription (RT) PCR and Western blotting. The CD97-knockdown cells were designated SGC-L/CD97-kd. Nonsilencing miRNA was used as the control.

### Fractionation of conditioned medium

The supernatant of tumor cells cultured in serum-free medium for 24 h was collected and centrifuged (10 min at 300*g* followed by 20 min at 16,500*g*) to pelletize the cells and debris. The supernatant was filtered through 0.20-μm filters to remove particles larger than 200 nm and stored as conditioned medium. Briefly, exosomes were pelletized by ultracentrifugation (Beckman Coulter, Fullerton, CA, USA) of the conditioned medium at 120,000*g* for 70 min at 4 °C; the vesicle-depleted supernatant was termed the soluble fraction, which contains a highly adhesive subfraction [14]. The pelletized exosomes were washed in phosphate-buffered saline (PBS) and pelletized by ultracentrifugation at 120,000*g* for 70 min. The exosome pellets were resuspended in PBS and stored at −80 °C until use. The total protein concentration of the exosomes was measured with an enhanced bicinchoninic acid protein assay kit (Beyotime, Nantong, China).

### Electron microscopy

Exosomes were resuspended in 1 % glutaraldehyde in PBS (pH 7.4) and pipetted onto Formvar carbon-coated electron microscopy grids. The sample was stained with 2 % phosphotungstic acid for 1 min and dried under an electric incandescent lamp for 10 min before it was viewed under a Tecnai 10 transmission electron microscope (Philips, Amsterdam, Netherlands). Exosome size was measured with use of the scale bar.

### Total RNA extraction and RT-PCR

Total RNA was extracted with TRIzol (Life Technologies). RT-PCR was performed according to the manufacturer’s protocol. Table S2 lists the primers used.

### Western blotting

Total proteins were resolved and transferred to poly(vinylidene difluoride) membranes (Millipore, Darmstadt, Germany). After they had been blocked with 5 % nonfat milk, the membranes were incubated (overnight, 4 °C) with anti-CD97 (1:5000, Abnova, Taipei, Taiwan), anti-CD55 (1:5000, Abcam, Cambridge, MA, USA), anti-CD44v6 (1:1000, Abcam), anti-EGF receptor (EGFR; 1:10000, Abcam), anti-human EGFR2 (HER2; 1:200, Abcam), anti-heat shock protein 70 (1:1000, Abcam), anti-CD9 (1:1000, Abcam), and anti-β-actin (1:5000, Bio-Ker, Gessate, Italy) antibodies. Then, the membranes were incubated with horseradish peroxidase conjugated goat anti-mouse immunoglobulin G or goat anti-rabbit immunoglobulin G (1:5000; Bio-Ker) and developed with an enhanced chemiluminescence kit (Millipore, Darmstadt, Germany).

### Immunohistochemistry

Formalin-fixed, paraffin-embedded sections (4 μm) were subjected to pretreatment by heat-mediated antigen retrieval with sodium citrate buffer (pH 6) and incubated overnight at 4 °C with anti-CD97 (1:200; Abnova), anti-CD44v6 (1:200; Abcam), anti-CD31 (1:200; Abcam), anti-α_5_β_1_ (1:200; Abnova), anti-CD31 (1:400; Abnova), anti-epithelial cell adhesion molecule (EpCam; 1:400; Abnova), and anti-CD151 (1:400; Abnova) antibodies. The sections were incubated with horseradish peroxidase conjugated secondary antibody (1:2000) and counterstained with Mayer’s hematoxylin (Invitrogen, Grand Island, NY, USA).

### Statistical analysis

Statistical analysis was performed by Student’s *t* test and one-way analysis of variance. Bonferroni correction was applied for multiple comparisons, dividing the significance level by the number of tested variables. All experiments were performed at least in triplicate; the results are expressed as the mean ± standard deviation. A probability (*p*) of 0.05 or less was considered statistically significant.

## Results

### Cancer stem cell marker expression was higher in lymph nodes of gastric adenocarcinoma with lower degrees of differentiation

**Fig. 1:**
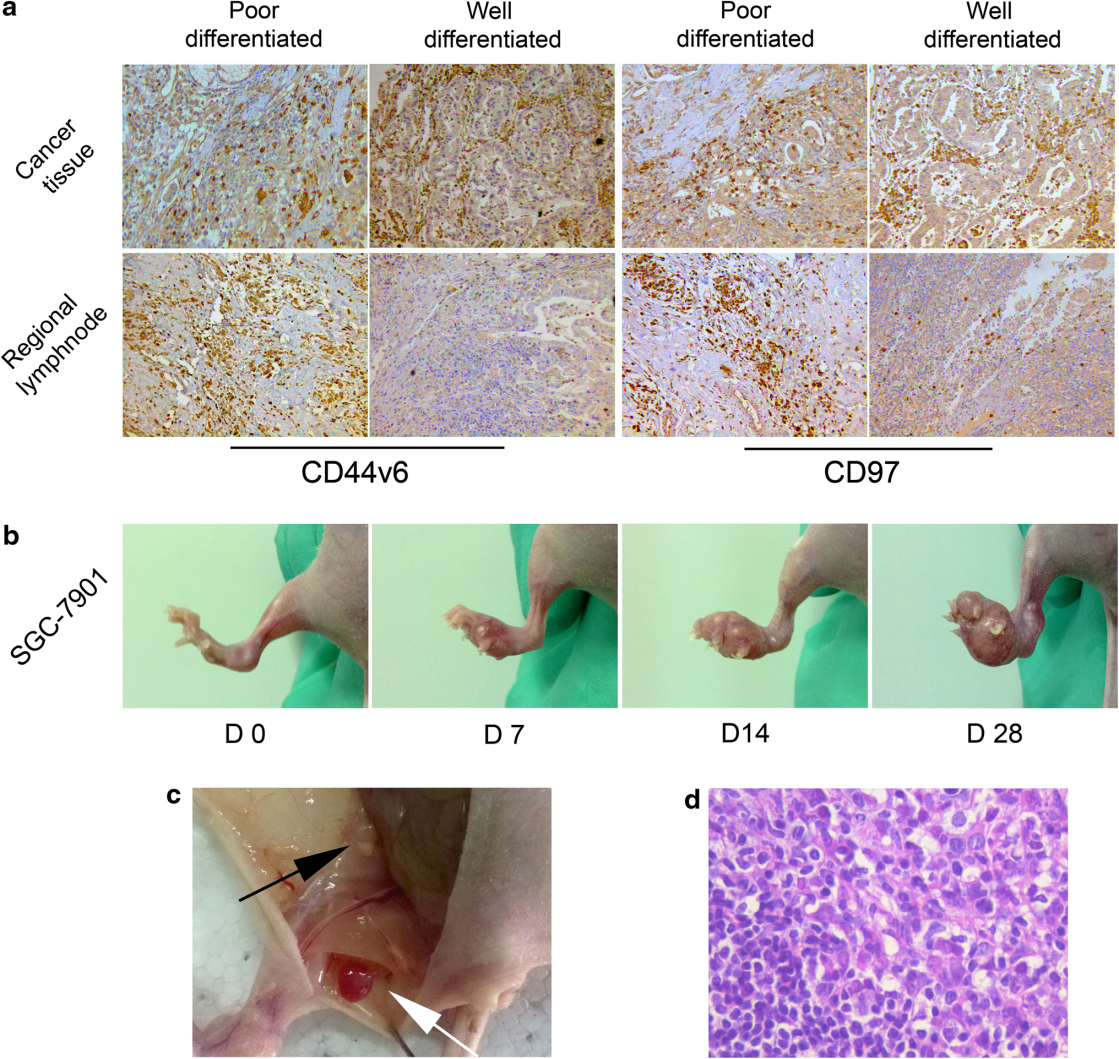
**a** Cancer tissues and regional lymph nodes of well-differentiated and poorly differentiated gastric adenocarcinoma showing high CD44v6 and CD97 expression (×200). Staining in the regional lymph nodes of poorly differentiated gastric cancer was stronger than for well-differentiated tumor samples (×200). **b** Local tumor growth 0, 7, 14, and 28 days after SGC-7901 cell injection. **c** Enlarged popliteal lymph node (*white arrow*) and inguinal lymph node (*black arrow*) on day 28. **d** Hematoxylin and eosin stained popliteal lymph node showing metastatic tumor cells on day 28 after SGC-7901 cell injection (×400). *D* day

Gastric adenocarcinoma tissue samples at the invasive front of the tumor mass and perigastric lymph nodes (along the greater gastric curvature) were harvested for evaluation. All regional lymph nodes were microscopically confirmed as nonmetastatic. Immunohistochemical investigation revealed that CD44v6 and CD97 expression was high in all tumor tissues independently of their degree of differentiation. There was higher CD44v6 and CD97 expression in the regional lymph nodes from poorly differentiated gastric cancer tissues with higher metastatic potential than in lymph nodes from well-differentiated gastric cancer (Fig. 1a). These findings suggest that, in addition to its association with tumor differentiation and invasion, CD97 may participate in premetastatic niche formation.

### Footpad lymph node metastasis mouse model and SGC-L cell generation

Human SGC-7901 cells were cultured, harvested, and injected into the footpads of BALB/c nu/nu mice. On days 7, 14, and 28 after injection, the mice were killed, and the footpad draining lymph nodes (popliteal and inguinal) were collected (Fig. 1b, c). Apart from the gradually increasing tumor volume in situ, enlarged popliteal and inguinal lymph nodes were detected on day 28. Hematoxylin–eosin staining revealed metastatic tumor cells in the popliteal lymph nodes (Fig. 1d), confirming the footpad lymph node metastasis mouse model. To generate a subpopulation of gastric cancer cells with higher metastatic ability, lymph nodes with metastatic tumor cells were filtered through fine gauze, cultured, and the harvested metastatic tumor cells were reinjected into the footpads of the mice. After 28 days, the metastatic tumor cells were harvested from the draining lymph nodes. The separation procedure was repeated four times, and we obtained the SGC-L subpopulation.

### Proliferative, migratory, and invasive abilities were stronger in SGC-L cells

**Fig. 2:**
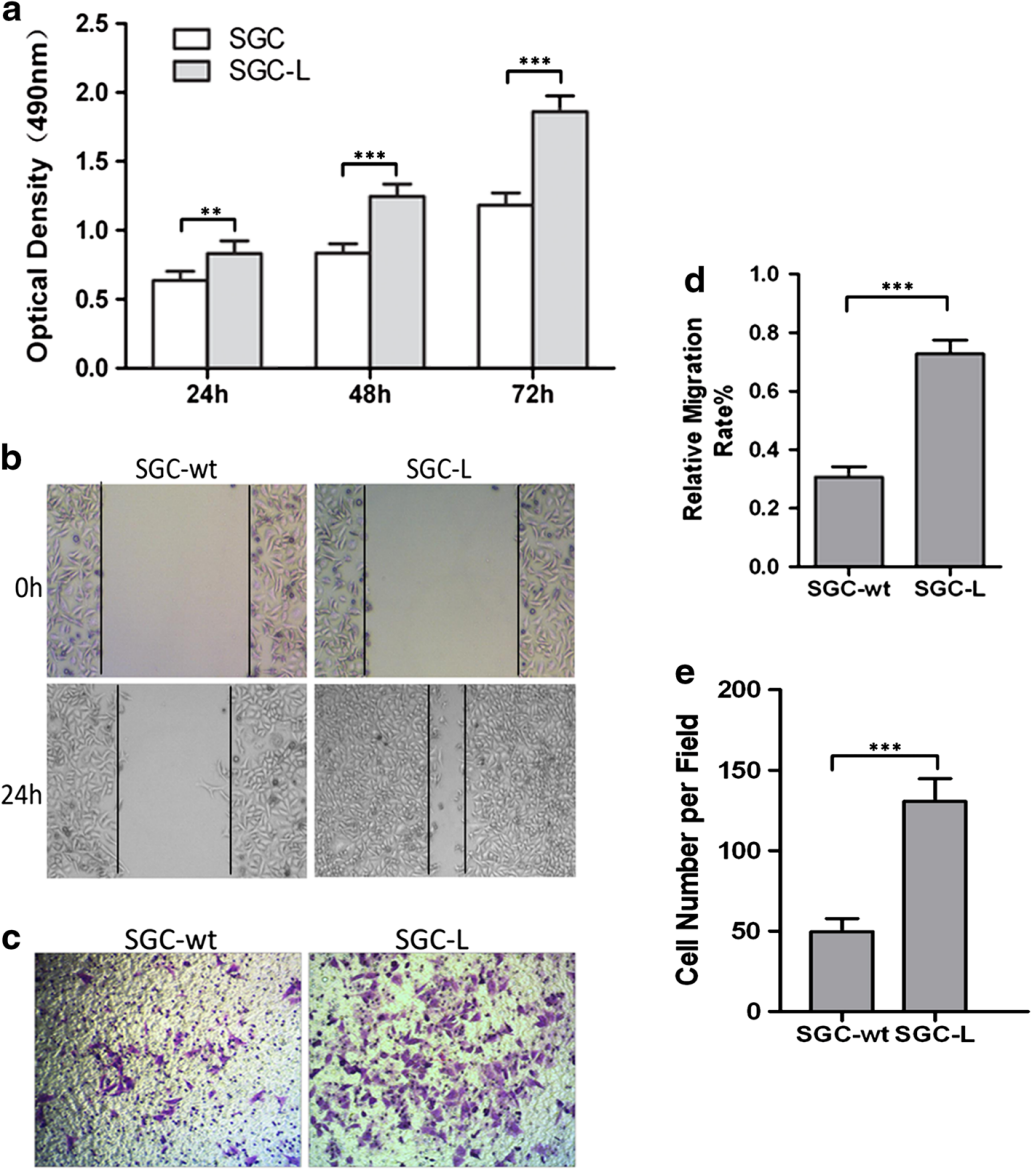
SGC-L cell proliferative, migratory, and invasive abilities are stronger than those of SGC-7901 cells. **a** 3-(4,5-Dimethylthiazol-2-yl)-5-(3-carboxymethoxyphenyl)-2-(4-sulfophenyl)-2*H*-tetrazolium (MTS) assay revealing significantly higher proliferative ability of SGC-L cells as compared with SGC-7901 (*SGC*) cells. **b** Scratch wound assay assessment of SGC-7901 (*SGC-wt*) and SGC-L cell motility. **d** The relative migration rate was calculated as the proportion of the mean distance of cell regrowth to the mean distance between the scratch borders. **c**, **e** Invasion assay revealing significantly increased numbers of SGC-L cells as compared with SGC-7901 (*SGC-wt*) cells. ** *p* < 0.01, *** *p* < 0.001

The MTS assay revealed that SGC-L cell proliferation rates were 60 % higher than those of SGC-7901 cells (Fig. 2a). In the scratch wound assay, continuous movement was observed in both groups, but the SGC-L-cell-free scratch region was smaller (42 %) than that of SGC-7901 cells, indicating the enhanced motility of the SGC-L cells (Fig. 2b, d). The migration assay demonstrated more migrated SGC-L cells than SGC-7901 cells, confirming the scratch wound assay results (data not shown). The invasion assay revealed two times more transmigrated SGC-L cells than SGC-7901 cells, demonstrating the increased invasive ability of SGC-L cells (Fig. 2c, e). These observations demonstrate the enhanced tumor-promoting potential of SGC-L cells as compared with SGC-7901 cells.

### Tumor-related cell membrane receptor expression was enhanced in SGC-L cells

**Fig. 3:**
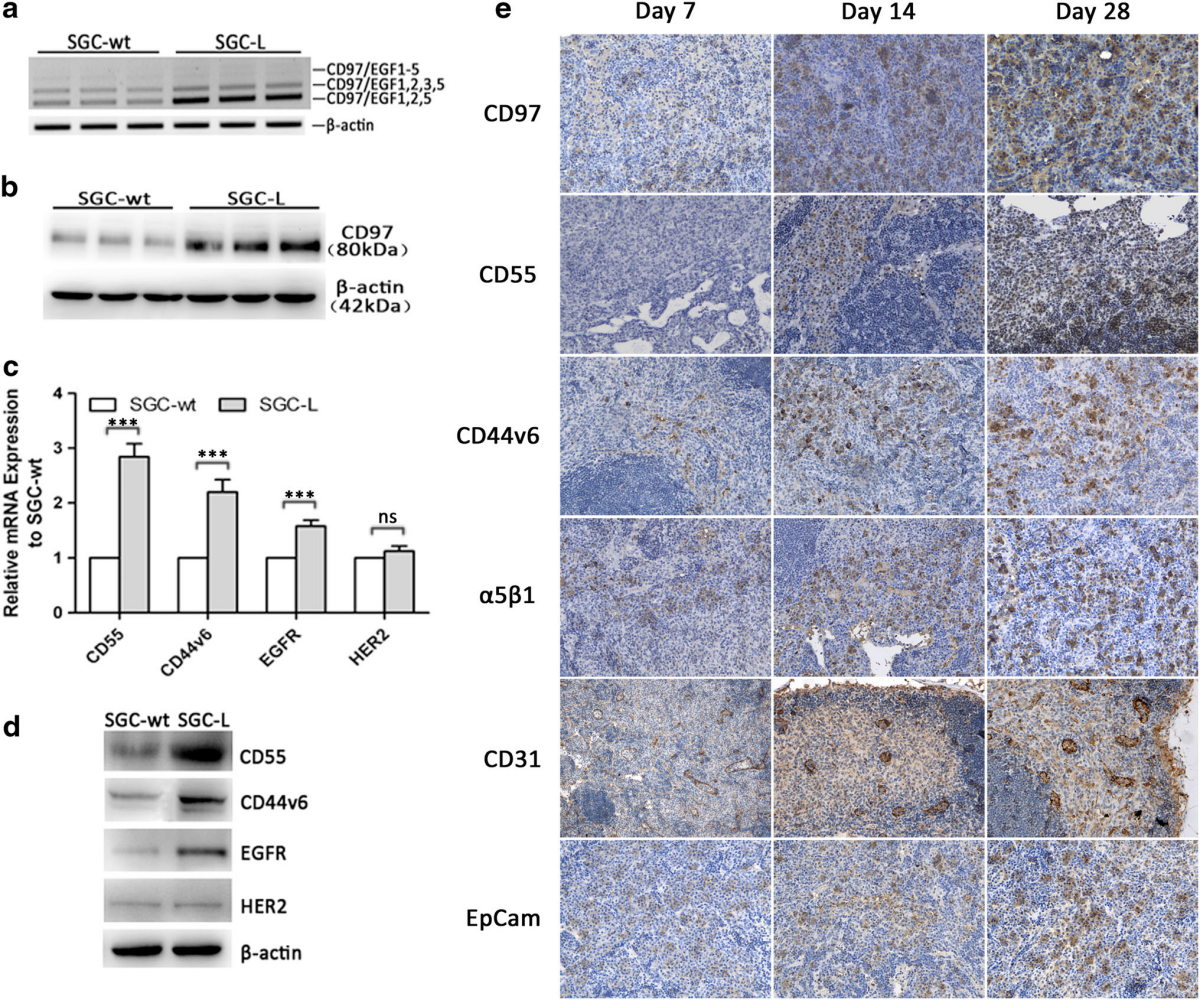
Alteration of transcript and protein expression in the SGC-L cell line established from SGC-7901 cells. **a** Reverse transcription PCR and **b** Western blot investigation of CD97 expression. SGC-L cell CD97 levels were higher than those of SGC-7901 (*SGC-wt*) cells. **c** Analysis of relative transcript expression revealing significantly increased CD55, CD44v6, and epidermal growth factor receptor (*EGFR*) messenger RNA (*mRNA*) expression in SGC-L cells as compared with SGC-7901 (*SGC-wt*) cells. There were no significant changes for human EGFR2 (*HER2*). **d** Western blotting revealing significantly higher protein expression. **e** Immunohistochemistry of nonmetastatic inguinal lymph nodes showing altered CD97, CD55, CD44v6, α_5_β_1_, CD31, and epithelial cell adhesion molecule (*EpCam*) expression on days 7, 14, and 28 after tumor cell injection (×200). *Three asterisks* *p* < 0.001, *ns* not significant

Enhanced CD97 expression in gastric cancer is associated with more evident malignancy [4]. Here, the proliferative, migratory, and invasive abilities and CD97 expression markedly differed in the SGC-7901 and SGC-L cells. To verify our hypothesis, we evaluated CD97 expression in SGC-7901 and SGC-L cells by RT-PCR and Western blotting. SGC-L cells expressed more CD97 transcripts (Fig. 3a) and protein (Fig. 3b) than SGC-7901 cells. Further, we tested the expression of CD97-related factors (membrane receptors): CD55 (a CD97 ligand), CD44v6 (a cancer stem cell marker), and two potential tumor therapeutic markers, EGFR and HER2. There was higher mRNA (Fig. 3c) expression of CD55 (2.9-fold), CD44v6 (2.2-fold), and EGFR (1.6-fold) in SGC-L cells than in SGC-7901 cells, as well as higher protein expression (Fig. 3d). The enhanced membrane receptor expression suggested that, in addition to the tumor-promoting role of CD97, other related membrane receptors or an enriched membrane receptor domain may enhance SGC-L cell invasive ability. Nonmetastatic inguinal lymph nodes harvested on day 28 after injection for immunohistochemical evaluation (Fig. 3e) revealed gradually concentrated CD97, CD55, CD44v6, α_5_β_1_, CD31, and EpCam expression in the draining lymph nodes on days 7, 14, and 28, suggesting the participation of these receptors in premetastatic niche formation.

### Generation of transfectants with stable knockdown of CD97 small isoform and identification of tumor-derived exosomes

**Fig. 4:**
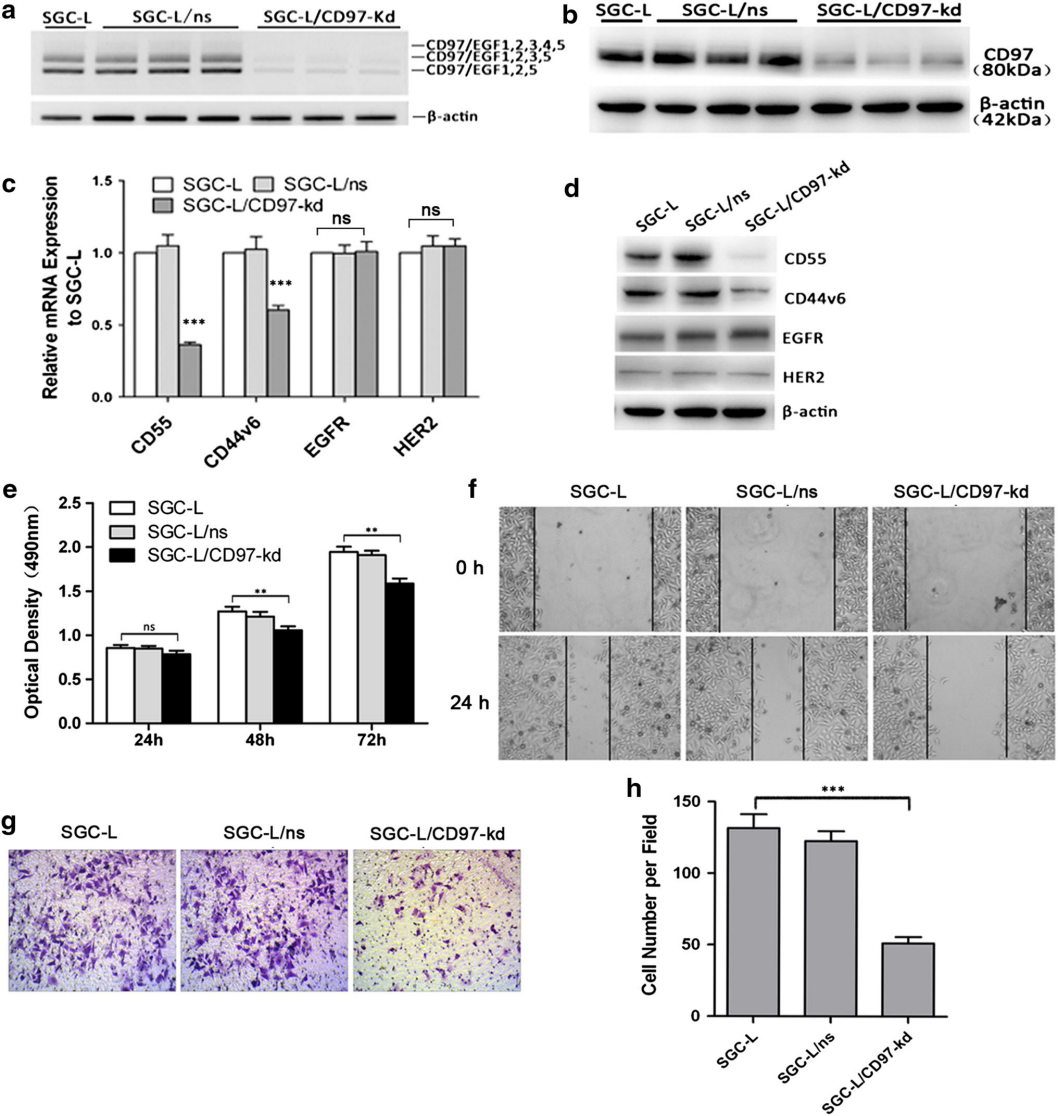
Transfectants with stable CD97 knockdown. **a** Reverse transcription PCR analysis was performed on SGC-L cells, nonsilencing microRNA clones (*SGC-L/ns)*, and SGC-L/CD97-knockdown (*SGC-L/CD97-kd*) clones; reference, β-actin. **b** Western blot detection of the approximately 80-kDa CD97 protein in total SGC-L, SGC-L/ns, and SGC-L/CD97-kd cell extracts. **c** CD97 knockdown dramatically decreased relative CD55 and CD44v6 messenger RNA (*mRNA*) expression in SGC-L/CD97-kd cells as compared with SGC-L or SGC-L/ns cells. Epidermal growth factor receptor (*EGFR*) and human EGFR2 (*HER2*) levels were not significantly affected. **d** Decreased CD55 and CD44v6 protein expression in SGC-L/CD97-kd cells as compared with the corresponding controls. **e** 3-(4,5-Dimethylthiazol-2-yl)-5-(3-carboxymethoxyphenyl)-2-(4-sulfophenyl)-2*H*-tetrazolium (MTS) assay revealing significantly decreased proliferation rates in SGC-L/CD97-kd clones at 48 and 72 h as compared with the corresponding controls. **f** Scratch wound assay and **g**, **h** Matrigel invasion assay showing significantly decreased motility and invasive ability, respectively, of SGC-L/CD97-kd clones as compared with SGC-L or SGC-L/ns controls (×200). *Two asterisks* *p* < 0.01, *three asterisks* *p* < 0.001, *ns* not significant

To investigate the role of CD97 in premetastatic niche formation and its possible mechanism, we generated CD97 small isoform (CD97iso) knockdown clones of SGC-L cells and isolated the tumor-derived exosomes. The CD97iso silencing efficiency was verified by RT-PCR and Western blotting. There was significant CD97iso knockdown and a parallel decrease of the levels of other CD97 isoforms (transcript and protein level) in the SGC-L/CD97-kd group as compared with the control (Fig. 4a, b). CD97 silencing led to significantly decreased levels of CD55 and CD44v6 as compared with the corresponding controls. However, CD97 knockdown did not affect EGFR and HER2 expression (Fig. 4c, d).

We used proliferation, migration, and invasion assays to investigate whether CD97 knockdown affected the behavior of the cells with high metastatic potential. The proliferative (Fig. 4e), migratory (Fig. 4f), and invasive (Fig. 4g, h) abilities of the SGC-L/CD97-kd clone were significantly decreased as compared with those of the control group.

**Fig. 5:**
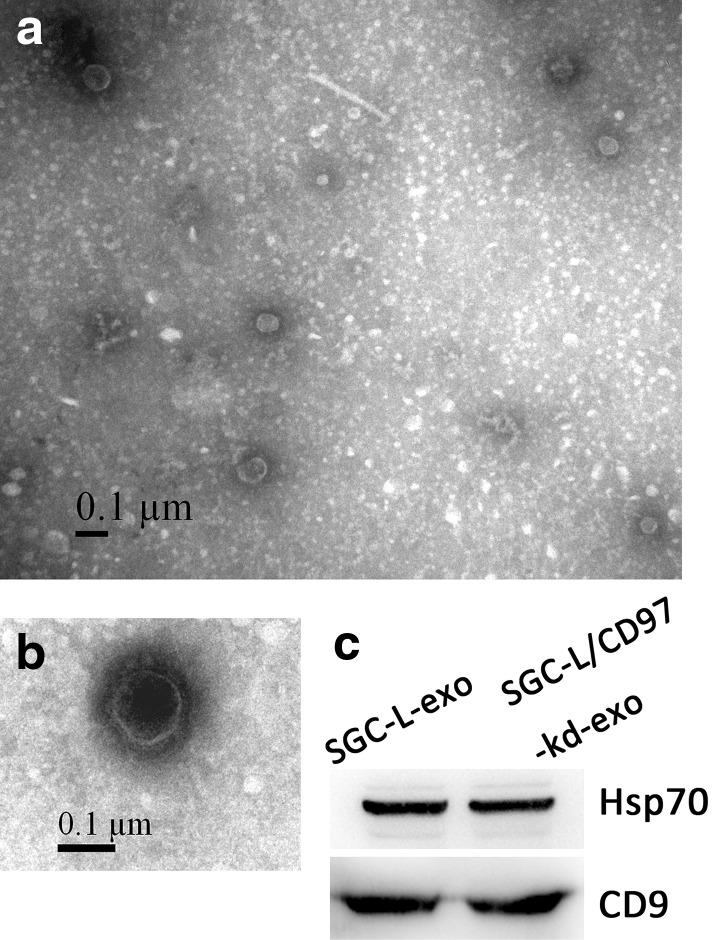
Identification of tumor-derived exosomes. **a**, **b** Electron micrographs of SGC-L exosomes (*SGC-L-exo*) and SGC-L/CD97-knockdown exosomes (*SGC-L/CD97-kd-exo*). **c** Western blotting analysis of exosomal proteins from SGC-L-exo and SGC-L/CD97-kd-exo cells. Equal amounts of exosomal lysate proteins were separated by 10 % sodium dodecyl sulfate–polyacrylamide gel electrophoresis, transferred to poly(vinylidene difluoride) membranes, and immunoblotted with anti-heat shock protein 70 (*Hsp70*) and anti-CD9 antibodies

On the basis of their unique size and density, the exosomes released by the SGC-L and SGC-L/CD97-kd cells were isolated and observed under an electron microscope. The exosomes appeared as small, closed, 30–100-nm-wide vesicles bound by a lipid bilayer, which was consistent with the reported size of exosomes [8]. We also detected the exosomal markers heat shock protein 70 and CD9 in the membrane vesicles (Fig. 5), which confirmed our successful isolation of tumor-derived exosomes [15, 16].

### SGC-L-cell-derived exosome and soluble fraction involvement and promotion of tumor cell proliferation and invasion in vitro

To support the hypothesis that exosomes, and the soluble fraction, promote long-distance metastasis and premetastatic niche formation, we separated exosomes and the soluble fraction from the conditioned medium by ultracentrifugation.

**Fig. 6:**
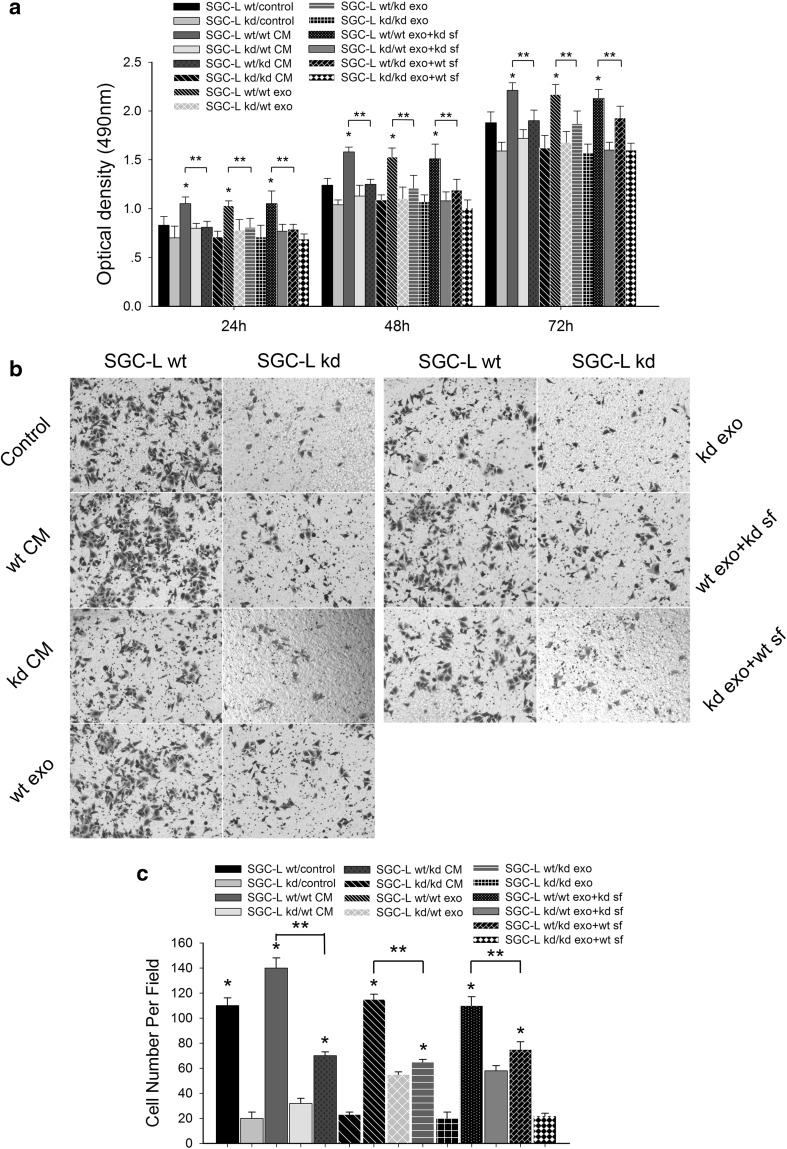
The impact of tumor-cell-derived conditioned medium, exosomes, and soluble fractions on cell proliferation and invasion. **a** Optical density (490 nm) of SGC-L and SGC-L/CD97-knockdown (SGC-L/CD97-kd) cells incubated with tumor-cell-derived conditioned medium (*CM*), exosomes (*exo*), or soluble fraction (*sf*) at 24, 48, and 72. *One asterisk* *p* < 0.01 versus control medium, *two asterisks* significant difference between SGC-L (*SGC-L wt*) and SGC-L/CD97-kd (*SGC-L kd*) conditioned medium and fractions (*exo*, *sf*). **b**, **c** Invasion assay of SGC-L and SGC-L/CD97-kd cells (×200). *One asterisk* significant difference versus SGC-L/CD97-kd cells, *two asterisks* significant difference between SGC-L (*SGC-L wt*) and SGC-L/CD97-kd (*SGC-L kd*) conditioned medium (*CM*) and fractions (*exo*, *sf*)

SGC-L cells were co-cultured with SGC-L-derived or SGC-L/CD97-kd-derived conditioned medium, exosomes, or the soluble fraction in vitro (Fig. 6). As expected, the SGC-L-derived conditioned medium dramatically enhanced cell proliferation (20 % increase) and invasion (30 % increase) as compared with the control medium. Such effects were observed, although less pronounced, when SGC-L/CD97-kd cells were incubated with SGC-L-derived conditioned medium. SGC-L/CD97-kd-derived conditioned medium impaired cell proliferation and did not influence SGC-L cell invasion. The findings confirm that SGC-L-derived conditioned medium promotes cell invasion and is involved in distant tumor metastasis.

To examine which conditioned medium components accounted for the metastasis-promoting effects, we isolated and investigated exosomes and the soluble fraction from SGC-L and SGC-L/CD97-kd cells. Similarly to the conditioned medium, SGC-L exosomes significantly promoted SGC-L (25 % increase) and SGC-L/CD97-kd (15 % increase) cell proliferation, albeit with less efficacy than that of conditioned medium. SGC-L/CD97-kd exosomes and soluble fraction did not affect cell invasive ability. However, co-culture of the cells with the SGC-L cell component fractions (i.e., SGC-L exosomes plus SGC-L/CD97-kd soluble fraction, or SGC-L soluble fraction plus SGC-L/CD97-kd exosomes) also enhanced cell proliferation and invasion. It appears that accelerated proliferation and enhanced invasion require both the soluble fraction and exosomes, and exosomes are mainly responsible for these effects. We investigated the effects of exosomes at 0, 50, 100, 200, and 400 μg/mL on cellular proliferation with the MTS assay. SGC-L exosomes increased SGC-L and SGC-L/CD97-kd cell proliferation dose dependently (data not shown).

### SGC-L-cell-derived conditioned medium and exosomes promoted SGC-L/CD97-kd cell lymphatic metastasis and modulated protein expression within premetastatic nodes

**Fig. 7:**
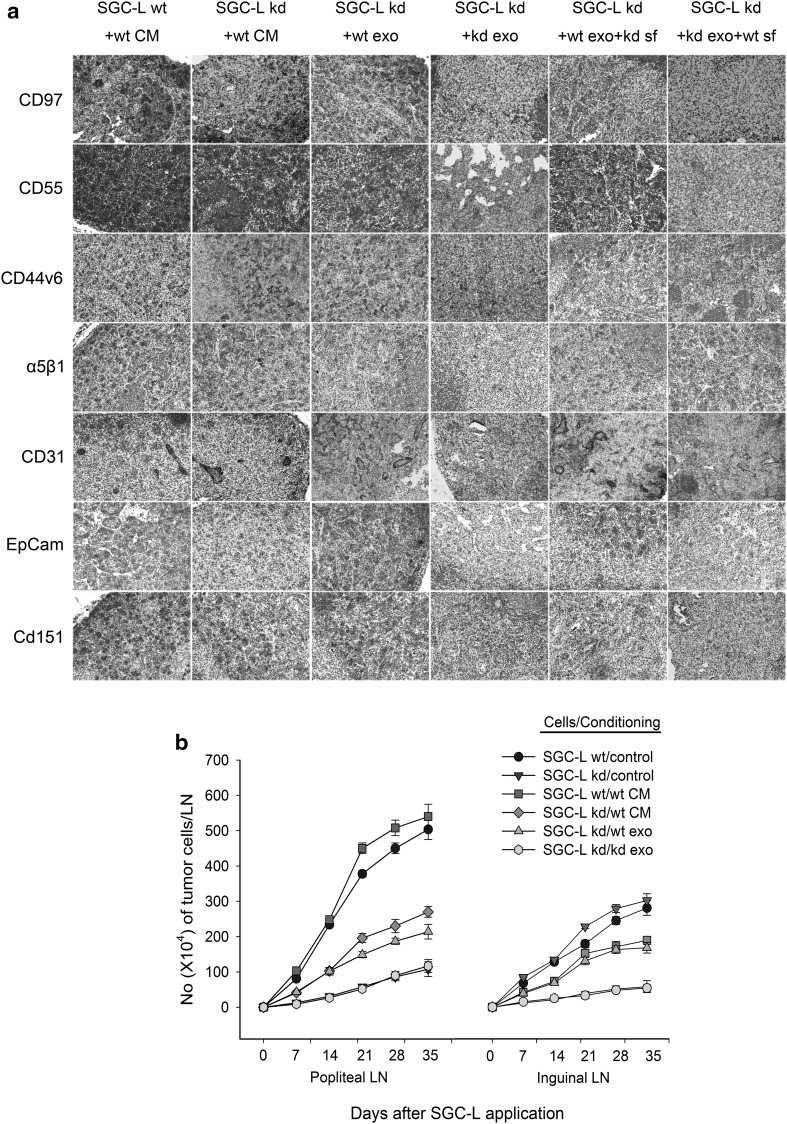
The impact of tumor cell conditioned medium/exosomes on metastasis. **a** Immunohistochemiical investigation of CD97, CD55, CD44v6, α_5_β_1_, CD31, epithelial cell adhesion molecule (*EpCam*), and CD151 expression in inguinal lymph nodes on day 35 (×200). **b** C4.4A staining and fluorescence-activated cell sorting of metastatic tumor cells within inguinal lymph nodes (*LN*) on days 7, 14, 21, 28, and 35

To evaluate the role of SGC-L or SGC-L/CD97-kd exosomes in lymph node metastasis, we established a footpad lymph node metastatic model. SGC-L or SGC-L/CD97-kd conditioned medium or exosomes were injected into the footpads of BALB/c nu/nu mice for five consecutive days before and twice weekly after tumor cell implantation. On days 7, 14, 21, 28, and 35 days after implantation, we collected the popliteal and inguinal lymph nodes and counted the metastatic tumor cells stained with C4.4A . SGC-L/CD97-kd cells treated with SGC-L/CD97-kd exosomes developed only several metastatic tumor cells (Fig. 7b), whereas SGC-L exosomes and conditioned medium had 60 % and 85 % increase of metastatic tumor cells, respectively, which confirmed the metastasis-promoting role of SGC-L exosomes.

CD55, CD44v6, α_5_β_1_, CD31, EpCam, CD151, and CD97 expression was increased in SGC-L cells treated with SGC-L conditioned medium. In contrast, the protein expression of these molecules was significantly decreased in SGC-L/CD97-kd cells treated with SGC-L/CD97-kd exosomes . The weak expression was rescued by SGC-L exosomes and was partly elevated by the SGC-L soluble fraction (Fig. 7a). The findings confirm that CD97 not only participates in premetastatic niche formation, but is also coexpressed and interacts with other membrane receptors.

## Discussion

We used a mouse footpad lymph node metastasis model and the highly lymphatic metastatic SGC-L cell line to demonstrate that CD97iso promotes gastric cancer cell metastasis exosome dependently. Furthermore, we demonstrated that CD97, assisted by the soluble fraction, plays a pivotal role in premetastatic niche formation.

Metastatic niche formation in the premetastatic organ plays an essential role in tumor metastasis [1]. Although many molecules, including CD44v6 [17], c-Met [18], urokinase-type plasminogen activator receptor [19], CD97 [7], CD9 [20], CD151 [21], CD11b [14], D6.1A [20], CD13 [22], CD49 [23], CD104 [24], EpCam [25], and claudin 7 [26], participate in this process, the recruitment incentive of these molecules to the metastatic site and the mechanism of long-distance communication with the local tumor remain unknown. Exosomes deliver proteins, mRNA, and miRNA to recipient targets distant from the local tumor, and are excellent messenger candidates for long-distance signal transduction [27]. We found that exosomes from gastric cancer cells lacking CD97iso were less effective promoters of lymphatic metastasis and that local tumor development was clearly impaired. Consequently, it appears that exosomes are the key metastatic factor in gastric carcinoma, and their participation in metastasis and niche formation depends on CD97iso expression.

CD97 is a member of the EGF–seven transmembrane subfamily that belongs to class B G-protein-coupled receptors, and is produced as three isoforms containing three (EGF1,2,5), four (EGF1–3,5), or five (EGF1–5) EGF domains [3, 28]. There are three CD97 ligands: chondroitin sulfate, a glycosaminoglycan that binds specifically to the CD97 large isoform (CD97/EGF1–5) and affects cell attachment [29]; α_5_β_1_ integrin, which binds via the Arg-Gly-Asp motif in the stalk region of CD97 [30]; and CD55, a glycosylphosphatidylinositol-linked membrane protein that binds specifically to CD97iso [31]. Differing from chimpanzees, most CD97 transcripts in humans encode the smallest isoform [32], suggesting that total CD97 mainly presents CD97iso characteristics. Consequently, CD97iso is a suitable subject for investigation.

CD55, or decay-accelerating factor, plays a crucial role in protecting the cell membrane from complement-mediated attack and lysis [33]. Early-stage gastric carcinomas are easily exposed to the complement attack environment, and tumor cells expressing CD55 protein can escape complement lysis [34]. Herein, CD97iso knockdown decreased CD55 expression, suggesting that CD97-mediated CD55 knockdown disrupts the membrane attack complex and exposes carcinoma cells to complement attack. The enhanced CD97 expression promoted local tumor progression, supporting our findings.

CD97 knockdown also decreased the levels of CD44, which is expressed in several leukemia and carcinoma cell types and is a marker of cancer-initiating cells [35]. CD44v6 overexpression promotes metastasis formation by its associating with and altering cytoskeletal proteins, integrins, and tetraspanin-enriched microdomains [36]. Here, the CD97-mediated CD44v6 expression decrease affected tumor cell biological behavior and reduced tumor cell metastatic ability. These findings demonstrate the possible involvement of CD44-dependent signal pathway cross talk with CD97 in premetastatic niche formation.

Increasing evidence shows that tumor-derived exosomes promote tumor progression through various mechanisms [10, 37]. Koga et al. [38] reported that exosomes secreted by BT-474 human breast adenocarcinoma cells attach to cell surfaces and increase the proliferation of the secreting cells. In gastric carcinoma, tumor-derived exosomes promote tumor cell proliferation via phosphatidylinositol 3-kinase/Akt activation [39]. However, it has been speculated that not only exosomes participate in long-distance premetastatic niche formation. To prove this hypothesis, we established a footpad lymph node metastasis mouse model. Conditioned medium from the highly metastatic cells established in this model promoted cell proliferation and invasion independently of CD97 expression. In contrast, conditioned medium from SGC-L/CD97-kd cells exerted weak tumorigenic effects. These data demonstrate that CD97iso knockdown affects not only cell biological behavior, but also remotely influences target cells though microvesicle-mediated signal transduction.

We demonstrated the essential role of exosomes in promoting gastric carcinoma cell invasion and metastasis. In our studies, the exosomes obtained acted as autonomous units that increased cell metastatic ability and the transfer of cells to the draining lymph nodes. The soluble fraction, which contains so-called adhesive markers, did not induce cell metastatic ability sufficiently. Although the SGC-L/CD97-kd exosomes maintained a structure similar to that of the SGC-L exosomes, they could not induce the prometastatic effects. The mechanisms responsible for these actions are largely unknown, but the involvement and alteration of membrane protein expression are considerable. In addition to the increased CD97, CD55, and CD44v6 expression within the draining inguinal lymph nodes pretreated with SGC-L conditioned medium or exosomes, α_5_β_1_, CD31, CD151, and EpCam levels were subsequently elevated. Most of these proteins are involved in active tumor cell migration and are related to poor prognosis in breast cancer, pancreatic cancer, colorectal cancer, and non-small-cell lung cancer [21, 22, 25, 40].

Several features of pancreatic-cancer-initiating cells are mostly preserved in weakly metastatic ASML CD44v6-knockdown cells; the tetraspanins are unimpaired in ASML CD44v6-knockdown exosomes, which might contribute to their metastasis-supporting activity [17]. However, we found that tetraspanins, e.g., α_5_β_1_, were significantly downregulated in the premetastatic niche of the SGC-L/CD97-kd exosome group, which not only had no supporting effect but also displayed increased resistance to tumor cell invasion in vitro. These findings suggest that altered CD44 expression may partly alter tetraspanin expression. Tetraspanin expression alteration within the tetraspanin-enriched microdomain during premetastatic niche formation is not always CD44 related; it is also potentially related to CD97, and may be involved in CD97-induced granulocytosis [41]. However, the mechanism requires clarification. Moreover, there was statistically significantly greater vessel density (CD31) in the premetastatic lymph nodes, which was potentially due to the angiogenesis-stimulating effect of CD97/EGF1–5.

In summary, we used the footpad lymph node metastasis mouse model and conditioned medium fractionation to demonstrate that CD97 promotes gastric carcinoma cell proliferation, migration, and invasion in vitro and contributes to premetastatic niche formation via exosomes. Although exemplified in an animal model, the findings require validation to demonstrate their relevance in human cancer progression.

## Electronic supplementary material

Below is the link to the electronic supplementary material.

Supplementary material 1 (PDF 42 kb)
