# Supplementary material for: CD97 promotion of gastric carcinoma lymphatic metastasis is exosome dependent
Source: Gastric Cancer. 2015 Aug 2;19:754–66. doi: 10.1007/s10120-015-0523-y (PMC4906076; doi:10.1007/s10120-015-0523-y)
Supplement: Supplementary file 1 — Supplementary material 1 (PDF 42 kb) [file 10120_2015_523_MOESM1_ESM.pdf]

**Supplementary Table S1** miRNA candidate and negative control sequences for CD97.

| oligo | Sequence (5’ -3’ ) |
|---|---|
| **1F** | TGCTGATGACATTCTGGATGGTGACCGTTTTGGCCACTGACTGACGGTCACCACAGAATGTCAT |
| **1R** | CCTGATGACATTCTGTGGTGACCGTCAGTCAGTGGCCAAAACGGTCACCATCCAGAATGTCATC |
| **2F** | TGCTGTATCTTCAAGGTTTGAGAGCAGTTTTGGCCACTGACTGACTGCTCTCACCTTGAAGATA |
| **2R** | CCTGTATCTTCAAGGTGAGAGCAGTCAGTCAGTGGCCAAAACTGCTCTCAAACCTTGAAGATAC |
| **3F** | TGCTGAAGAAAGTAGAGCTCCAGGCCGTTTTGGCCACTGACTGACGGCCTGGATCTACTTTCTT |
| **3R** | CCTGAAGAAAGTAGATCCAGGCCGTCAGTCAGTGGCCAAAACGGCCTGGAGCTCTACTTTCTTC |
| **4F** | TGCTGAAGATGAACAGGCCAAAGACCGTTTTGGCCACTGACTGACGGTCTTTGCTGTTCATCTT |
| **4R** | CCTGAAGATGAACAGCAAAGACCGTCAGTCAGTGGCCAAAACGGTCTTTGGCCTGTTCATCTTC |
| **Negative-F** | TGCTGAAATGTACTGCGCGTGGAGACGTTTTGGCCACTGACTGACGTCTCCACGCAGTACATTT |
| **Negative-R** | CCTGAAATGTACTGCGTGGAGACGTCAGTCAGTGGCCAAAACGTCTCCACGCGCAGTACATTTC |

**Supplementary Table S2** Primer sequences

| Primer name | | Sequence (5' - 3') |
|---|---|---|
| CD97 | Sense | actctgccgggagctgaaac |
| | Antisense | tggatggtgacctcggctga |
| CD55 | Sense | ttccaaggtcccaccaacag |
| | Antisense | accatcaacacccctggttc |
| CD44v6 | Sense | ggcaacagatggcatgaggg |
| | Antisense | agtggtatgggaccccccactgggg |
| EGFR | Sense | ggcctaagatcccgtccatc |
| | Antisense | tggctttcggagatgttgtc |
| HER2 | Sense | cacccaaagccaacaaagaa |
| | Antisense | cacatcctccaggtagctcatc |
| β-actin | Sense | aggctggtcctcctatcgtt |
| | Antisense | tctgggtaagagcccaggtt |
